# IL-33-induced neutrophil extracellular traps degrade fibronectin in a murine model of bronchopulmonary dysplasia

**DOI:** 10.1038/s41420-020-0267-2

**Published:** 2020-05-04

**Authors:** Rui Jin, Junjie Xu, Qianqian Gao, Xiaonan Mao, Jiao Yin, Keyu Lu, Yan Guo, Mingshun Zhang, Rui Cheng

**Affiliations:** 1 Department of Neonatal Medical Center, Children’s Hospital of Nanjing Medical University, 210008 Nanjing, China; 2 NHC Key Laboratory of Antibody Technique, Department of Immunology, Nanjing Medical University, 211166 Nanjing, China

**Keywords:** Immune cell death, Innate immunity

## Abstract

Bronchopulmonary dysplasia (BPD) is the leading cause of chronic lung disease in preterm neonates. Extracellular matrix (ECM) abnormalities reshape lung development, contributing to BPD progression. In the present study, we first discovered that the ECM component fibronectin was reduced in the pulmonary tissues of model mice with BPD induced by lipopolysaccharide (LPS) and hyper-oxygen. Meanwhile, interleukin-33 (IL-33) and other inflammatory cytokines were elevated in BPD lung tissues. LPS stimulated the production of IL-33 in alveolar epithelial cells via myeloid differentiation factor 88 (MyD88), protein 38 (p38), and nuclear factor-kappa B (NF-κB) protein 65 (p65). Following the knockout of either IL-33 or its receptor suppression of tumorigenicity 2 (ST2) in mice, BPD disease severity was improved, accompanied by elevated fibronectin. ST2 neutralization antibody also relieved BPD progression and restored the expression of fibronectin. IL-33 induced the formation of neutrophil extracellular traps (NETs), which degraded fibronectin in alveolar epithelial cells. Moreover, DNase-mediated degradation of NETs was protective against BPD. Finally, a fibronectin inhibitor directly decreased fibronectin and caused BPD-like disease in the mouse model. Our findings may shed light on the roles of IL-33-induced NETs and reduced fibronectin in the pathogenesis of BPD.

## Introduction

Despite escalating efforts to manage bronchopulmonary dysplasia (BPD), the incidence and mortality of BPD in preterm neonates still pose a great challenge^1^. The extracellular matrix (ECM) functions as a scaffold that guides pulmonary development, and ECM composition and structure continuously evolve as the lung matures^2^. In the ECM, fibronectin attaches to cells via integrins that regulate migration and differentiation^3^. In the classical (old) form of BPD, increased fibronectin may contribute to an excessive reparative process and pulmonary fibrosis^4,5^. The expression of fibronectin in lung development varies at different phases of maturation, coinciding with alveolar septa formation^6^. In the pseudoglandular stage, fibronectin around developing airways may be essential for branching morphogenesis and alveolar epithelial cell differentiation^7^. We speculate that inadequacy of fibronectin may hinder the alveolus formation in the premature lung. The mechanisms of fibronectin fine-tuning in the pathogenesis of BPD are largely unresolved.

Recently, the roles of interleukin-33 (IL-33) in BPD pathogenesis have attracted much attention. IL-33 is a novel member of the IL-1 cytokine family^8^. Upon cell injury, full-length IL-33 is released from the nucleus and cleaved into its mature form, which binds its receptor suppression of tumorigenicity 2 (ST2) and initiates a type 2 immune response^9,10^. Unlike mature IL-33, full-length IL-33 predominately strengthens the inflammatory response independent of ST2^11^. In the peripheral blood of BPD patients, mature IL-33 and soluble ST2 were found to be increased^12^. Moreover, elevated IL-33 in the peripheral blood is a predictable marker for BPD diagnosis and disease severity^13^. As expected, increased IL-33 was associated with retarded lung development in a murine model of BPD induced by hyperoxia^14^. In neonatal transgenic mice, the overexpression of full-length IL-33 in lung epithelial cells caused an elevation in mature IL-33 levels, leading to alveolar abnormalities similar to those in BPD^15^. All of these studies highlighted the importance of IL-33 in the pathogenesis of BPD.

The influence of IL-33 signaling on pulmonary fibrosis may be controversial. On the one hand, IL-33, with its receptor ST2, was found to aggravate bleomycin-induced lung fibrosis^16^. On the other hand, IL-33 amplified the formation of neutrophil extracellular traps (NETs)^17^, fibrous structures composed of DNA, histones and various proteases^18^. NETs are closely associated with ventilator-induced lung injury^19^ and other lung diseases^20^. Armed with proteases, NETs may cleave the ECM component laminin^21^. We hypothesized that IL-33 induced the formation of NETs, which degraded fibronectin and arrested lung maturation. To test this hypothesis, we established a mouse model of BPD with perinatal exposure to lipopolysaccharide (LPS) and hyper-oxygen^22^. In this BPD model, disease severity was evaluated by IL-33 or ST2 deficiency or blockade with ST2 neutralization antibody. DNase was administered to explore the roles of NETs in BPD pathogenesis. Finally, a fibronectin inhibitor was used to directly assess the roles of fibronectin in lung development. The present study evidenced that LPS caused the release of IL-33 from epithelial cells, which promoted NETs formation and decreased fibronectin in pulmonary tissues. Meanwhile, IL-33 deficiency or ST2 deficiency ameliorated the disease severity of BPD, highlighting the essential roles of IL-33/ST2 signaling pathway in the BPD pathogenesis. DNase and ST2-neutralizing antibody improved the lung development in the murine model of BPD, shedding new light on BPD therapy.

## Results

### Decreased fibronectin and elevated IL-33 in the early stage of BPD

**Fig. 1:**
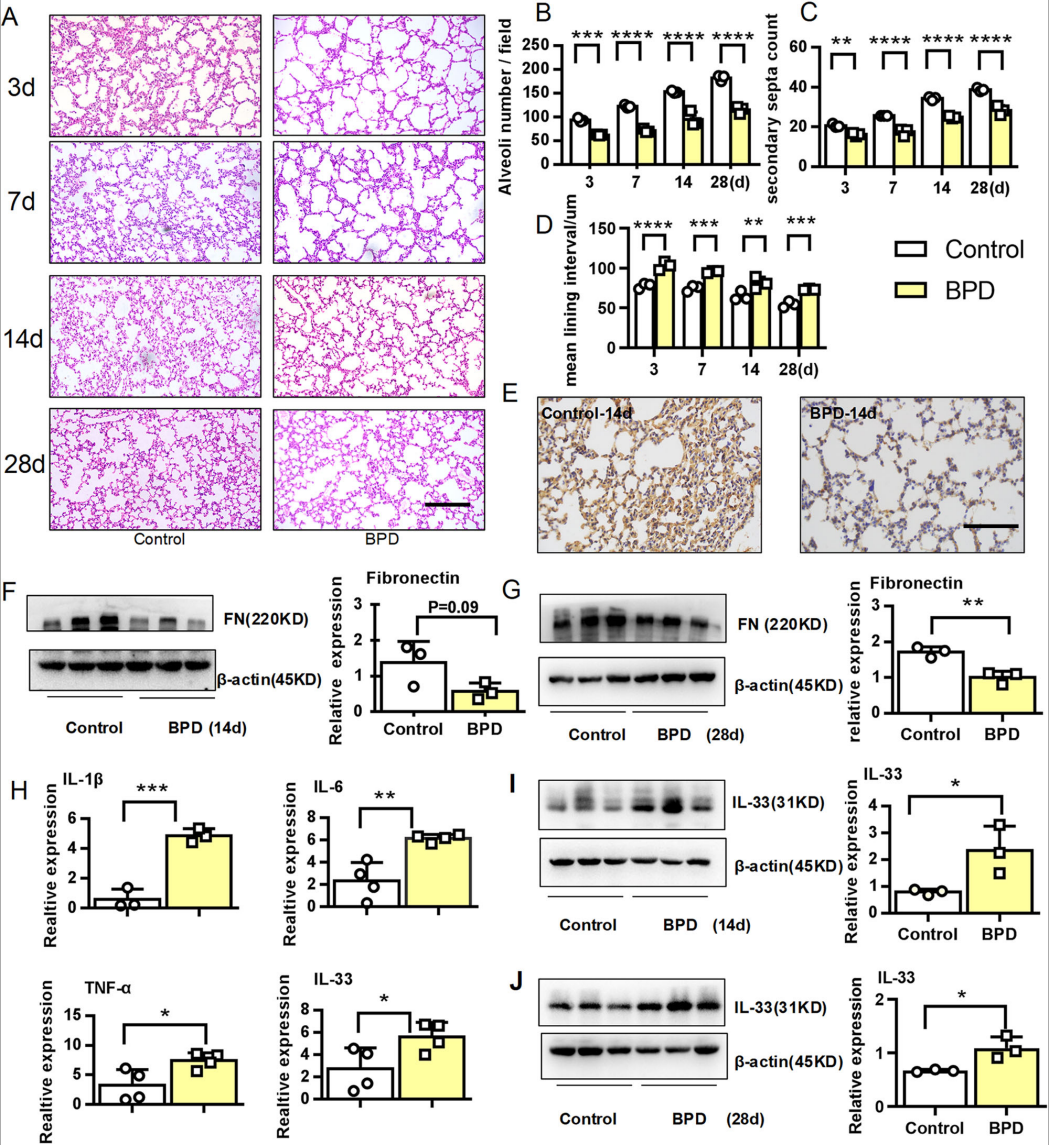
Fibronectin was decreased, while IL-33 was increased in the early stage of BPD. E14–15d pregnant dams were intraperitoneally injected with 75 µg/kg LPS, and newborn mice were exposed to 65% hyper-oxygen to establish BPD model. **a**–**d** Lung morphometry was analyzed by H&E staining of tissue samples from surviving pups at day 3, 7, 14, and 28 post birth. Scale bars = 200 μm. **e** Representative immunohistochemical analysis of fibronectin in pulmonary tissues at day 14 post birth. Scale bars, 100 μm. **f**–**g** Western blotting analysis of fibronectin expression in pulmonary tissues at day 14 and 28 post birth. **h** IL-1β, IL-6, TNF-α, and IL-33 mRNA expression in pulmonary tissues at day 14 post birth. **i**–**j** IL-33 protein expression in pulmonary tissues at day 14 and 28 post birth. Three to four mice were included in the control and BPD group at each time point. All experiments were repeated at least three times. *P < 0.05, **P < 0.01, ***P < 0.001.

The combination of perinatal inflammation and postnatal hyperoxia was used to trigger severe BPD-like lung disease in rodents (21, 22). Accordingly, we established the murine model of BPD with perinatal LPS and postnatal hyperoxia. Compared with the control group, histological analysis demonstrated arrested alveolar development, with a significantly decreased number of large, simplified alveoli and sparse secondary septation in the BPD group. In contrast, mean lining interval in the BPD group was significantly increased (Fig. 1a–d). The expression of fibronectin in the lung tissues from BPD group was significantly reduced at 14 days after birth (P14) (Fig. 1e, f) and P28 (Fig. 1g). Meanwhile, the inflammatory factors IL-1β, IL-6, and TNF-α were elevated at the gene level in the BPD group, as reported in a previous publication (Fig. 1h)^22^, and the expression of IL-33 was significantly increased in the lung tissues from the BPD group at P14 (Fig. 1h, i) and P28 (Fig. 1j). In short, we found that IL-33 was notably elevated, while fibronectin was significantly decreased in the lung tissues from a dual-hit model of BPD at the early stage of disease.

### LPS directly induced IL-33 expression in epithelial cells

**Fig. 2:**
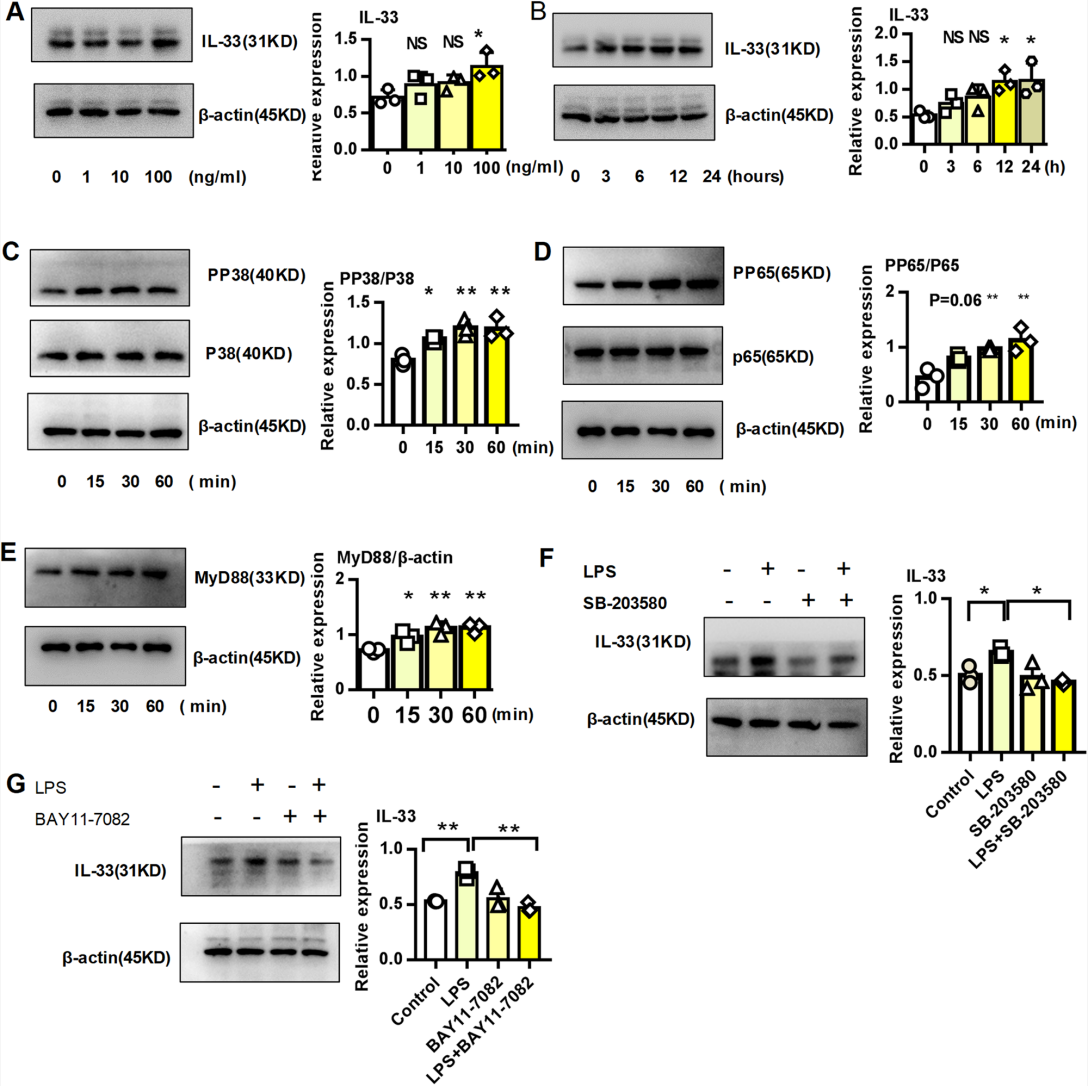
LPS directly induced IL-33 expression in epithelial cells in vitro. **a** Dose-dependent expression of the IL-33 protein in MLE-12 cells stimulated with LPS. **b** Time-dependent expression of the IL-33 protein in MLE-12 cells stimulated with 100 ng/ml LPS. **c**–**e** p38, p65, and MyD88 protein expression in MLE-12 cells stimulated with 100 ng/ml LPS. **f**–**g** IL-33 protein expression in MLE-12 cells stimulated with 100 ng/ml LPS in the presence or absence of the p38 inhibitor SB-203580 or the p65 inhibitor BAY 11–7082. All experiments were repeated at least three times. *P < 0.05, **P < 0.01, ***P < 0.001.

Intrauterine infection can lead to intrauterine inflammation, which not only triggers premature birth and serves as an important independent risk factor for premature birth but also plays an important role in the development of BPD^23,24^. IL-33 was significantly elevated in the pulmonary tissues from BPD group. Epithelial cells were major producers of IL-33. Therefore, we stimulated alveolar epithelial cells with LPS to simulate intrauterine infection and explore the origin of IL-33. As shown in Fig. 2a, b, LPS directly induced the expression of IL-33 on epithelial cells. The Toll-like receptor/myeloid differentiation factor 88 (MyD88) signaling pathway was essential for IL-33 expression after bacterial and viral infections^25^. Unsurprisingly, LPS activated MAPK protein 38 (p38), nuclear factor-kappa B (NF-κB) protein 65 (p65), and Myd88 (Fig. 2c–e) in alveolar epithelial cells. Furthermore, inhibitors of MAPK p38 (SB-203580) and NF-κB p65 (BAY 11–7082) rescued the expression of IL-33 in epithelial cells stimulated with LPS. Overall, these results indicated that LPS directly promoted the expression of IL-33 in lung epithelial cells through the p38 and p65 signaling pathways.

### IL-33 deficiency relieved the disease severity of BPD

**Fig. 3:**
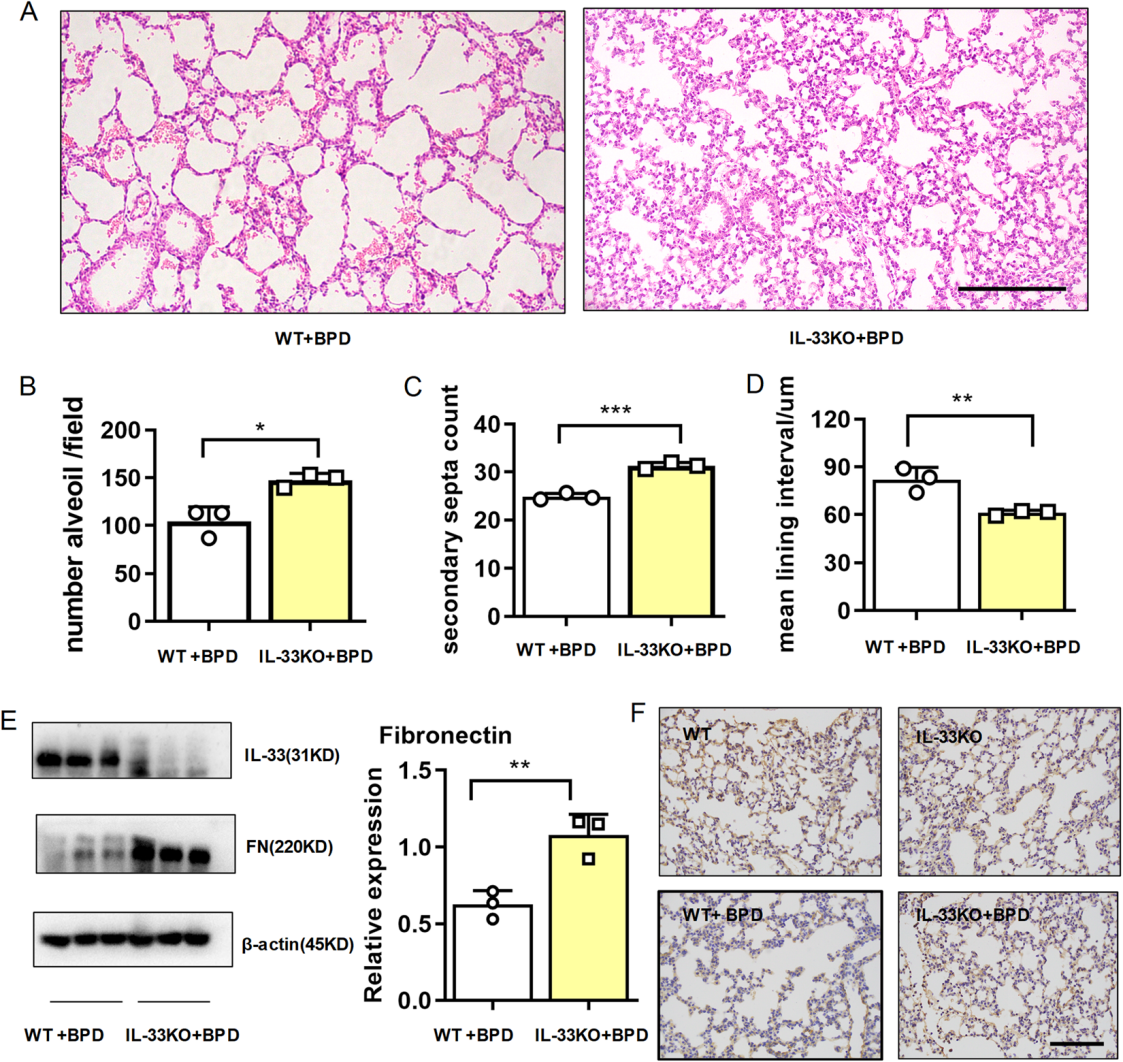
IL-33 deficiency relieved the disease severity of BPD. E14–15d pregnant dams (WT and IL-33 KO) were intraperitoneally injected with 75 µg/kg LPS, and newborn mice were exposed to 65% hyper-oxygen to establish BPD model. **a**–**d** Lung morphometry was analyzed by H&E staining of tissue samples from surviving pups at day 14 post birth. Scale bar, 200 μm. **e** IL-33 and fibronectin protein expression in pulmonary tissues at day 14 post birth. **f** Representative results of immunohistochemical analysis of fibronectin in pulmonary tissues at day 14 post birth. Scale bars, 100 μm. Three mice were included in the control and BPD group at each time point. All experiments were repeated at least three times.*P < 0.05, **P < 0.01, ***P < 0.001.

To explore the roles of IL-33 in BPD development, we developed a model of BPD in IL-33 deficient mice and compared that in the wild-type mice. As shown in Fig. 3a–d, histological analysis on day 14 demonstrated that IL-33 deficiency improved alveolar development, with significantly increased the number of alveoli and improved secondary septation. Conversely, the mean lining interval was remarkably diminished in the IL-33 deficient BPD-like mice. Using western blot assays (Fig. 3e) and immunohistochemistry (Fig. 3f), we discovered that the fibronectin was significantly higher in the IL-33 deficient BPD-like mice than in the wild-type BPD-like mice at 14 days after birth. Therefore, IL-33 deficiency ameliorated the disease severity of BPD, accompanied by elevated expression of fibronectin.

### IL-33 receptor deficiency improved BPD

**Fig. 4:**
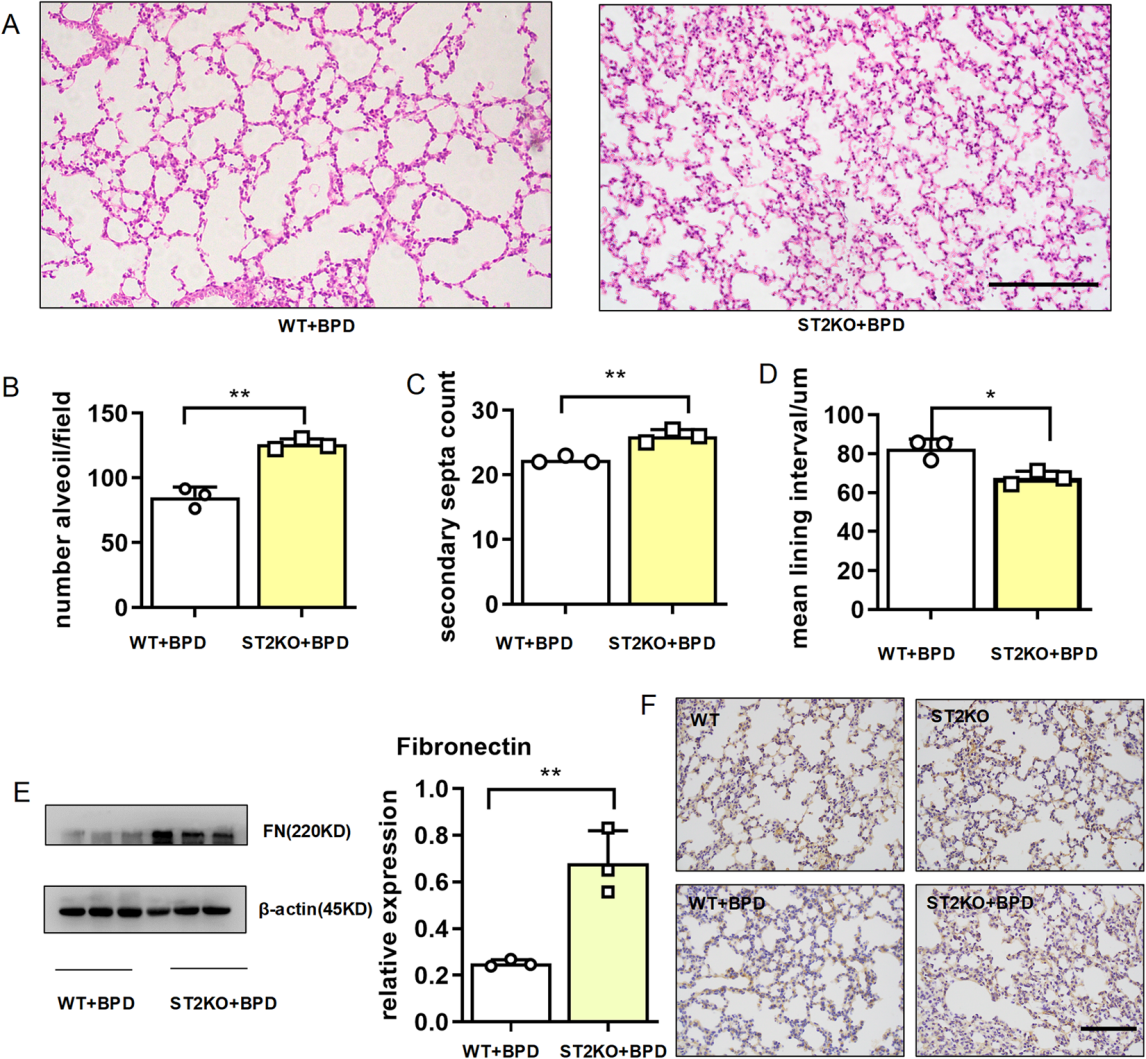
Knockout of the IL-33 receptor ST2 improved BPD. E14–15d pregnant dams (WT and ST2 KO) were intraperitoneally injected with 75 µg/kg LPS, and newborn mice were exposed to 65% hyper-oxygen to establish BPD model. **a**–**d** Lung morphometry was analyzed by hematoxylin and eosin (H&E) staining of tissue samples from surviving pups at day 14 post birth. Scale bars = 200 μm. **e** Fibronectin protein expression in pulmonary tissues at day 14 post birth. **f** Representative results of immunohistochemical analysis of fibronectin in pulmonary tissues at day 14 post birth. Scale bar, 100 μm. Three mice were included in the control and BPD group at each time point. All experiments were repeated at least three times. *P < 0.05, **P < 0.01, ***P < 0.001.

IL-33 may exert its effect with our without its receptor ST2. To determine the roles of ST2 in IL-33 mediated BPD pathogenesis, we subjected ST2-knockout mice to BPD modeling. Histological analysis of pulmonary tissues revealed that ST2 deficiency improved alveolar development, with a significantly increased number of alveoli and improved secondary septation, whereas the mean lining interval was remarkably diminished compared with that in the wild-type BPD-like mice (Fig. 4a–d). As observed in the IL-33 KO mice, knockout of the IL-33 receptor ST2 rescued the expression of fibronectin, which was validated using western blot assays (Fig. 4e) and immunohistochemistry (Fig. 4f). These results suggested that IL-33 promoted BPD development through the IL-33/ST2 axis.

### ST2-neutralizing antibody prevented BPD

**Fig. 5:**
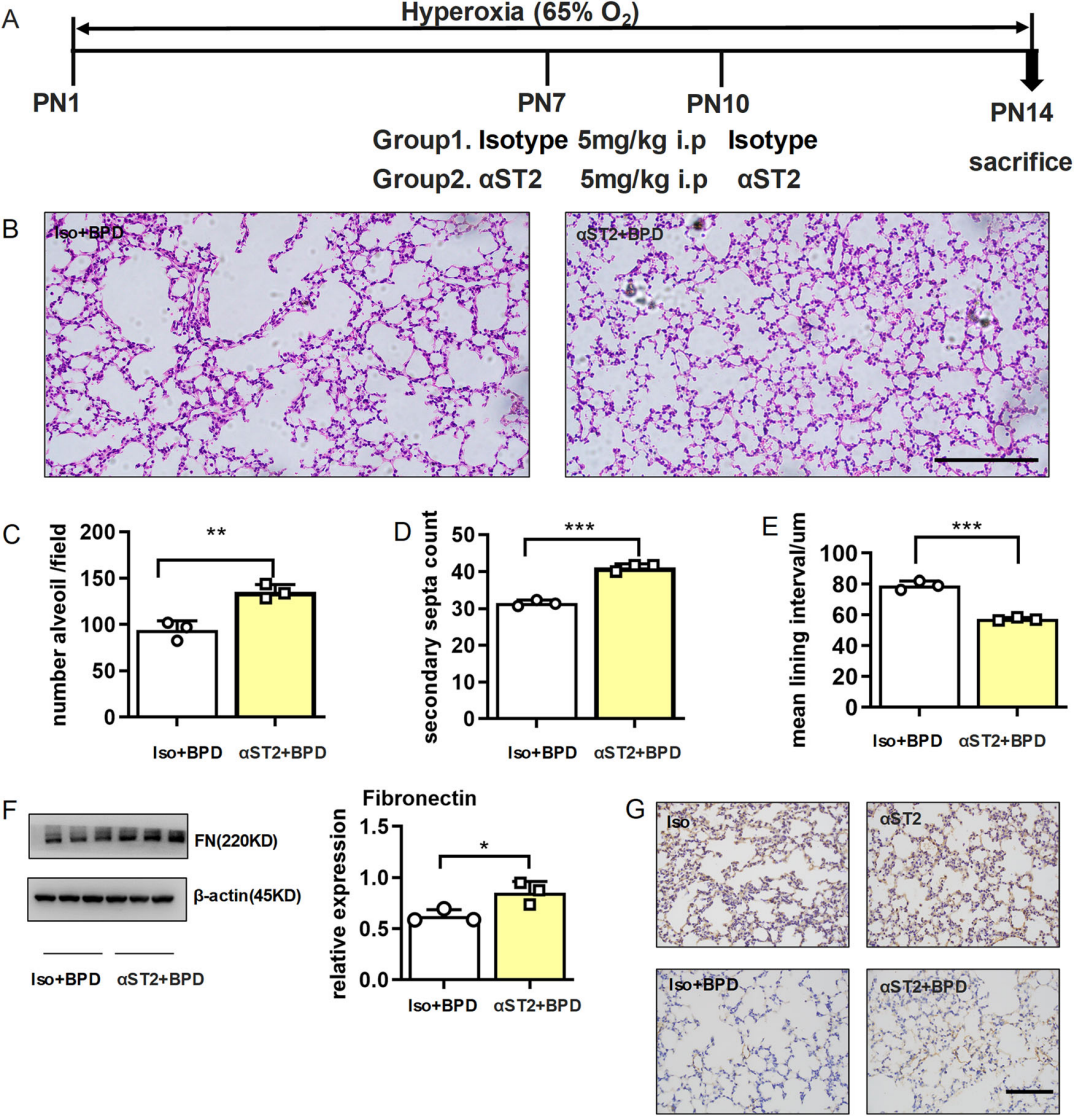
ST2 neutralization antibody relieved the disease severity of BPD. E14–15d pregnant dams were intraperitoneally injected with 75 µg/kg LPS, and newborn mice were exposed to 65% hyper-oxygen to establish BPD model. On the 7th and 10th day after birth, isotype control antibody or ST2-neutralizing antibody was simultaneously intraperitoneally injected into the pups, and the pups were sacrificed at day 14 post birth. **a** Design test plots for the use of isotype controls and ST2-neutralizing antibodies. **b**–**e** Lung morphometry was analyzed by H&E staining of tissue samples from surviving pups at day 14 post birth. Scale bars = 200 μm. **f** Fibronectin protein expression in pulmonary tissues at day 14 post birth. **g** Representative results of immunohistochemical analysis of fibronectin in pulmonary tissues at day 14 post birth. Three mice were included in the control and BPD group at each time point. All experiments were repeated at least three times. Scale bars, 100 μm. *P < 0.05, **P < 0.01, ***P < 0.001.

To facilitate their clinical application, we explored the therapeutic potential of ST2-neutralizing antibody in BPD. As depicted in Fig. 5a, pups were intraperitoneally injected with ST2 isotype control or ST2-neutralizing antibodies at 7 and 10 days after birth. Histological analysis on day 14 demonstrated that αST2 administration improved alveolar development, with a significantly increased number of alveoli and improved secondary septation and a remarkably diminished mean lining interval (Fig. 5b–e). Again, αST2 administration increased the expression of fibronectin in the lung tissues 14 days after birth (Fig. 5f, g). Collectively, these results suggested that αST2 was valuable for BPD therapy.

### DNase therapy alleviated BPD disease severity

**Fig. 6:**
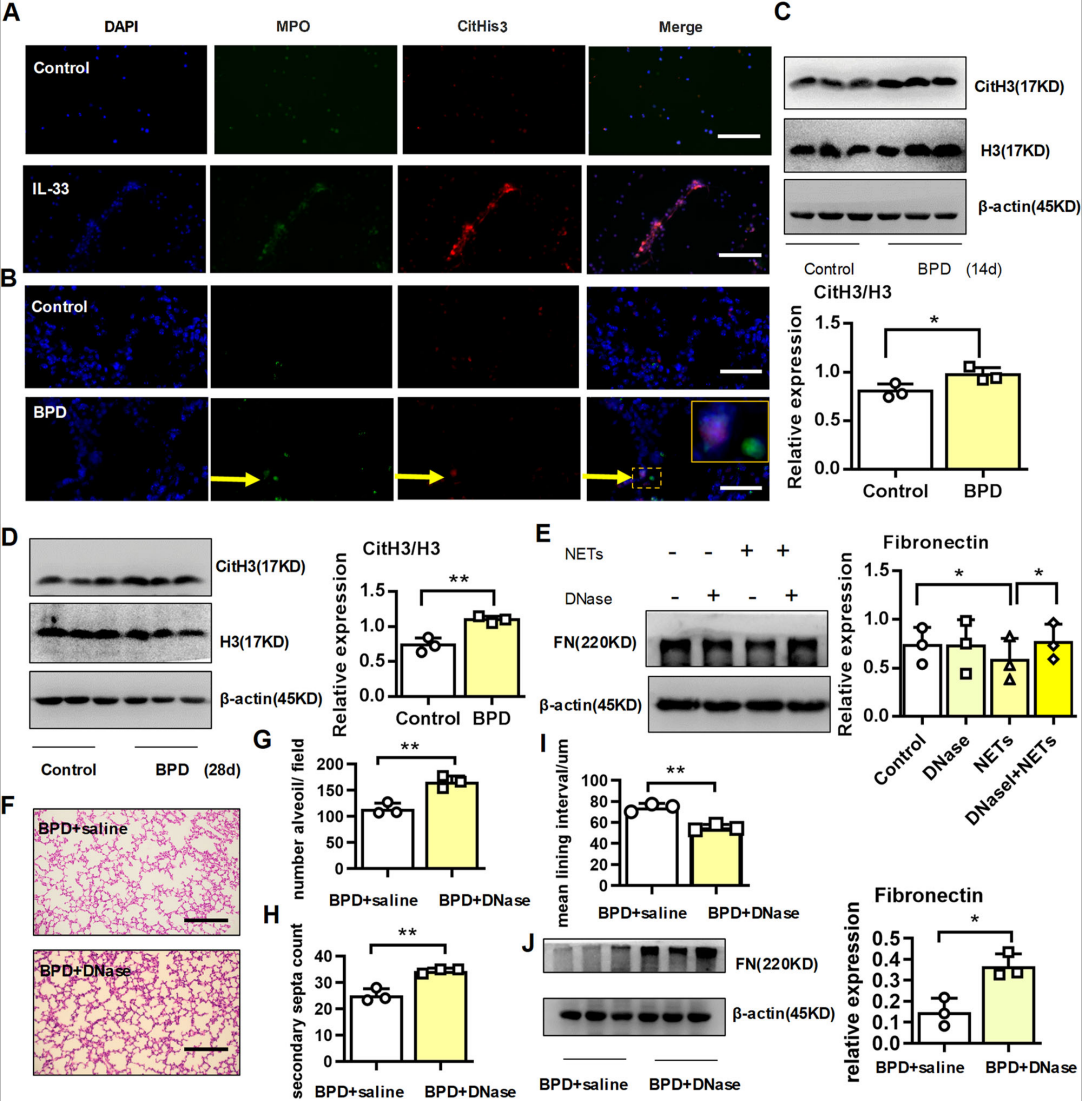
DNase alleviated the disease severity of BPD. **a**, **b** Representative immunofluorescence imaging of NETs in neutrophils after rIL-33 stimulation in vitro. **b** Representative immunofluorescence imaging of NETs in pulmonary tissues. Scale bars, 100 μm. **c**, **d** Cit-Histone 3 and Histone 3 protein expression in pulmonary tissues at day 14 and 28 post birth. **e** Fibronectin protein expression in MLE-12 cells stimulated with 100 ng/ml NETs in the presence or absence of 5 µM DNase. **f**–**i** Pregnant dams were injected with 75 μg/kg LPS at days 14–15 of gestation. After delivery, newborn mice were exposed to 65% hyper-oxygen to establish BPD model and received either saline or DNase (5 mg/kg) by twice-daily aerosol inhalation. Lung morphometry was analyzed by H&E staining of tissue samples from surviving pups at day 14 post birth. Scale bars = 200 μm. **j** Fibronectin protein expression in pulmonary tissues at day 14 post birth. Three mice were included in the control and BPD group at each time point. All experiments were repeated at least three times. *P < 0.05, **P < 0.01, ***P < 0.001.

Increased neutrophils in the peripheral blood^26^ and prolonged neutrophil influx into the lung^27^ indicated the disease severity of BPD. As previously reported^17^, we found that rIL-33 stimulated NETs formation (Fig. 6a). Elevated IL-33 from BPD suggested that NETs may develop in the pulmonary tissues of the BPD group. As expected, pulmonary NETs formed in BPD mice, as detected by the presence of extracellular DNA with citrullinated histone 3 and myeloperoxidase (MPO) using immunofluorescence imaging (Fig. 6b). Furthermore, the level of citrullinated histone H3 was significantly higher in the BPD group at P14 (Fig. 6c) and P28 (Fig. 6d). To investigate whether NETs played a role in the fibronectin expression of alveolar epithelial cells, we stimulated epithelial cells with purified NETs in vitro. As expected, NETs reduced the protein expression of fibronectin from epithelial cells. In contrast, DNase destructed the DNA skeleton of NETs and rescued the expression of fibronectin from NETs-treated-epithelial cells (Fig. 6e).

We further explored whether DNase reduced lung NETs formation and subsequently relieved BPD disease severity. To assess this hypothesis, we built a BPD model, and the pups then received the twice-daily aerosol inhalation of either saline or DNase. Histological analysis revealed that aerosol inhalation of DNase improved alveolar development, with significantly increased number of alveoli and improved secondary septation and remarkably diminished mean lining interval (Fig. 6f–i). DNase restored fibronectin expression in the pulmonary tissues from BPD-like mice (Fig. 6j). In brief, IL-33 promoted the development of NETs, and DNase alleviated BPD disease severity, accompanied with rescued fibronectin.

### Fibronectin inhibitor caused alveolar BPD-like disease

**Fig. 7:**
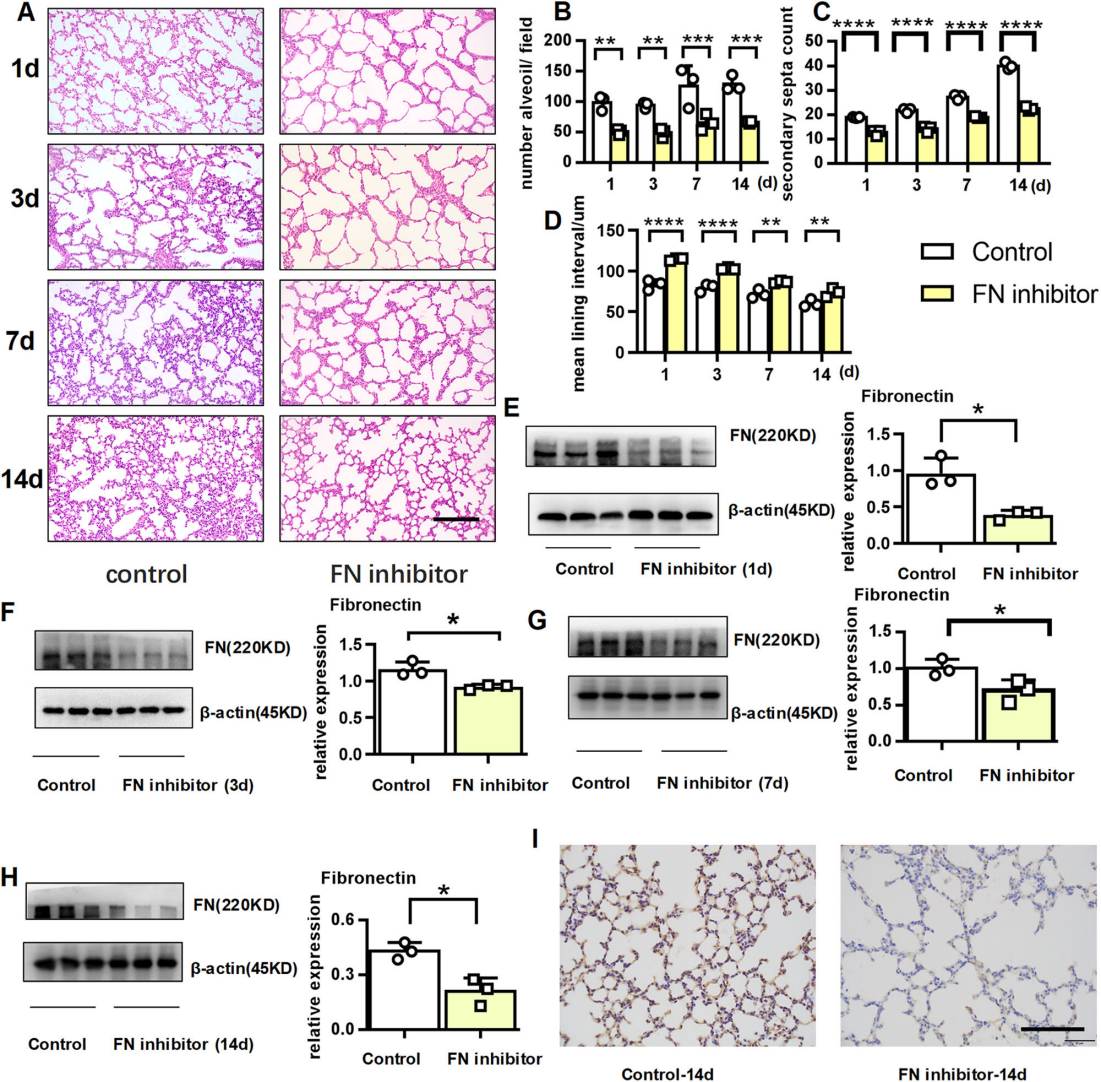
Fibronectin inhibitor caused BPD-like disease. E17d pregnant dams were injected with 5 mg/kg fibronectin inhibitor or an equal amount of saline. At day 3, 7, and 10 post birth, the pups were simultaneously given an intraperitoneal dose of 5 mg/kg fibronectin inhibitor or an equivalent amount of saline (control), and the pups were sacrificed on the 1st, 3rd, 7th, and 14th day. **a**–**d** Lung morphometry was analyzed by H&E staining of tissue samples from surviving pups at day 1, 3, 7, and 14 post birth. Scale bars = 200 μm. **e**–**h** Fibronectin protein expression in pulmonary tissues at day 1, 3, 7, and 14 post birth. **i** Representative immunohistochemical analysis of fibronectin in pulmonary tissues at day 14 post birth. Scale bars, 100 μm. Three mice were included in the control and BPD group at each time point. *P < 0.05, **P < 0.01, ***P < 0.001.

Thus far, we have demonstrated that fibronectin was reduced in BPD mice and that disease improvement was accompanied by fibronectin restoration. Therefore, we hypothesized that a reduction in fibronectin would directly lead to BPD. As expected, intraperitoneal injection of fibronectin inhibitor directly arrested alveolar development, as evidenced by a significantly decreased number of large, simplified alveoli, sparse secondary septation, and increased mean lining interval (Fig. 7a–d). Using western blot assays (Fig. 7e–h) and immunohistochemistry analysis (Fig. 7i), we ascertained that the expression of fibronectin was significantly lower in the mice treated with fibronectin inhibitor. These results further suggested that fibronectin insufficiency may contribute to BPD.

## Discussion

BPD is a chronic lung disease in preterm infants. Following exposure to pulmonary inflammation and other risk factors, lung development is prematurely arrested, characterized by reduced alveolarization and impaired gas exchange^28^. Prenatal exposure to LPS and post-birth hyper-oxygen ventilation simulated the clinical aspects of BPD, following which the pro-inflammatory cytokines IL-1β, IL-6, and TNF-α were significantly increased. Unexpectedly, fibronectin was reduced in lung tissues from the BPD group. Although classical (old) form of BPD was characterized by excessive pulmonary fibrosis, growing evidence also indicated the occurrence of new BPD^29^ in which alveolarization inhibition was accompanied by minimal fibrosis^30^. Indeed, fibronectin augmented airway branching, and anti-fibronectin blocked structural development of the fetal lung^31^, suggesting that fibronectin may function as an ECM to support airway sprouting. In line with the constructive role of fibronectin in lung maturation, a fibronectin inhibitor decreased fibronectin in the lung and caused BPD-like disease in our study. ECM disturbance in the developing lung was the hallmark of BPD pathology^2^. Defective expression^32–34^ or distribution^31^ of ECM components may lead to alveolar simplification and arrested lung development.

In our double-hit BPD model, IL-1β and IL-33 were significantly elevated in the neonatal lung. IL-1β exposure after birth caused BPD-like disease in infant mice^35^, and antibody blocking the IL-1 receptor diminished pulmonary inflammation and improved disease severity in a murine model of BPD developed by exposure to LPS and hyper-oxygen^22^. Similar to antibody blocking the IL-1 receptor, the neutralizing antibody against the IL-33 receptor, ST2, partially rescued lung development, preventing a decrease in alveolar number. Previously, IL-33-neutralizing antibody was found to suppress epithelial damage and pulmonary inflammation, benefiting lung development in BPD-like mice^14^. Therefore, IL-33 and ST2 may be targets in BPD therapy.

Exosomes from the neutrophils of BPD patients were found to contain elastase, which degraded ECM and led to BPD-like disease in neonatal mice^36^. NETs armed with neutrophil elastase and other enzymes are closely associated with chronic obstructive pulmonary disease^37^ and other pulmonary diseases. In the lung, NETs may cleave ECM via elastase and MMP-9^21^. We speculated that IL-33-induced NETs degrade fibronectin with elastase and other enzymes. In addition to fibronectin, elastin, and other ECM components may also be decomposed by NET-associated elastase^38^. As expected, elastin deficiency resulted in impaired lung airway branching^32^, and an elastase inhibitor was suggested to benefit lung growth in BPD^39,40^. In the present study, DNase alleviated BPD disease severity. A DNA backbone served as a scaffold for NETs; DNase degraded DNA and therefore diminished the functions of NETs^41^. DNase, which has been clinically approved for the treatment of cystic fibrosis^42^, warrants further examination in BPD therapy.

In summary, we demonstrated that fibronectin deficiency may contribute to BPD development, during which pulmonary tissue fibronectin was degraded by NETs. Elevated IL-33 in BPD induced the formation of NETs via ST2. Moreover, DNase degrading NETs and ST2 neutralization antibody blocking IL-33/ST2 signaling pathway may be potential alternatives in BPD therapy.

## Materials and methods

### Animals and ethics statement

Wild-type C57BL/6J mice (female and male) at 6–8 weeks of age were obtained from the Laboratory Animal Center of Yangzhou University (Yangzhou, China) and bred under specific pathogen-free conditions at Nanjing Medical University. IL-33-knockout or ST2-knockout mice with a C57BL/J background were obtained from Dr Hong Zhou (Department of Immunology, Nanjing Medical University). All animal protocols were reviewed and approved by the Animal Care and Use Committee of Nanjing Medical University (1910018).

### Animal model of BPD

Based on previous publications^22,43^, males and females were randomly paired, and the presence of a vaginal plug was used to designate embryonic (E) day 1 (E1). On E14, pregnant C57BL/6J dams randomly received an intraperitoneal (i.p.) injection of 75 μg/kg LPS (Sigma #L2880) or an equal volume of saline. Within 24 h after birth, pups and dams were randomized to a chamber through which we passed gas at 5 L/min with an FiO_2_ of 0.21 (room air) or 0.65 for a total of 3, 7, 14, or 28 days. The temperature (22 °C) and humidity (50–60%) were kept constant. To avoid oxygen toxicity in the dams and to eliminate maternal effects between groups, the nursing dams were rotated between their hyperoxic and room air litters every 24 h. All mice were maintained on a 12-h light–dark cycle. Mice were euthanized on days 3, 7, 14, and 28 by the injection of pentobarbital sodium (200 mg/kg i.p.).

To explore the roles o IL-33/ST2 signaling pathway in the BPD pathogenesis, IL-33-knockout or ST2-knockout mice were treated with LPS and 65% oxygen as described in the wild-type mice.

To examine the therapeutic potential of ST2 neutralization antibody, wild-type pups in the BPD model were randomly allocated to receive 5 µg/kg anti-ST2 antibody (MAB10041-SP, R&D) or isotype antibody (MAB0061, R&D) by i.p. injection on days 7 and 10.

To examine the roles of NETs and the therapeutic potential of DNase, wild-type pups in the BPD model were administered with 5 mg/kg DNase I (DN25–1G, Sigma) or an equal volume of saline via aerosol inhalation twice a day.

To study the direct role of fibronectin, pregnant dams at E17d received an i.p. injection of 5 μg/kg fibronectin inhibitor (CAS 91037–65–9, Cruz). After delivery, the pups were also randomly allocated to receive i.p. injections of 5 µg/kg fibronectin inhibitor every 3 days from day 3 until day 10. Saline administration was set as the control.

### Cell culture

The MLE-12 cell line was obtained from ATCC (CRL-2110, USA) and cultured according to the protocol provided by ATCC. MLE-12 cells were cultured in DMEM (HyClone, USA) with 10% fetal bovine serum (Lonsera, USA) and 1% penicillin/streptomycin (HyClone, USA) in a humidified atmosphere of 5% CO_2_ at 37 °C overnight. MLE-12 cells were treated with 0, 1, 10, or 100 ng/ml LPS (Sigma, USA) or an equal volume of phosphate-buffered saline (PBS) (HyClone, USA) as a control. When needed, NF-κB inhibitor BAY 11–7082 (Selleckchem, USA) or p38 MAPK inhibitor SB-203580 (Selleckchem, USA) were added to the medium at a final concentration of 5 µM. Cells were harvested 24 h later for further analysis.

### Neutrophil isolation

Mouse neutrophils were isolated from the bone marrow of tibias and femurs as described previously^44^. In brief, the mice were anesthetized, and the surface of each animal was sprayed with 70% ethanol. The muscles were removed from both legs, and the femur was separated from the tibia at the knee joint. The bone marrow cells were flushed using a sterile syringe filled with PBS containing 0.5% endotoxin-free bovine serum albumin (BSA) and 2 mM ethylenediaminetetraacetic acid and collected into a 50 ml conical tube through a 70 µm cell strainer. The bone marrow cells were collected by centrifugation at 500 × *g* for 5 min at 4 °C. The cell pellet was resuspended in 1 ml of RBC lysis buffer for 5 min. The cell suspension was washed and centrifuged to collect the cells. The cell pellet was resuspended in 200 µl of magnetic-activated cell sorting (MACS) buffer, and 50 µl of neutrophil biotin-antibody cocktail was added. Then, the cell suspension was mixed thoroughly and incubated for 10 min in the refrigerator at 4 °C. The cell suspension was washed and centrifuged to collect the cells. The cell pellet was resuspended in 400 µl of MACS buffer, and 100 µl of anti-biotin microbeads was added. Then, the cell suspension was mixed well and incubated for 15 min in the refrigerator at 4 °C. The cell suspension was washed and centrifuged to collect the cells, and the cell pellet was resuspended in 500 µl of MACS buffer. The cells were subsequently loaded onto a MACS buffer-equilibrated LS column (Miltenyi Biotec), which was washed three times with 3 ml of MACS buffer. The cells eluted from the LS column were harvested and used for further analysis.

### Lung sample preparation

Mice were sacrificed by the intraperitoneal injection of sodium pentobarbital and exsanguination by aortic transection. After aortic transection, a thoracotomy was performed, the right bronchus was ligated, and the right lungs were removed and snap frozen. The tracheas were cannulated, and the left lungs were inflated and fixed with 4% formalin (G1101, Servicebio, China) at a pressure of 25 cmH_2_O for ≥15 min. After equilibration, the left lungs were removed and fixed in 4% formalin overnight. Subsequently, lung tissues were cut into 5-μm-thick sections on a Leica model 2165 rotary microtome (Leica, Nussloch, Germany) and stained with hematoxylin and eosin (H&E) as previously described^45,46^.

### Lung H&E staining

Tissue sections were stained with H&E for morphometric analyses. To assess uniform and proportional samples from each lung, three nonoverlapping photomicrographs in different sections were captured at ×200 magnification with a microscope (model BX-53, Olympus Optical) under identical lighting conditions and optical settings by an investigator blinded to the grouping. Three images per animal were analyzed and averaged using research-based digital image analysis software (ImageJ, JAVA). The analytical methods used to determine the number of alveoli and secondary septa and mean lining interval were described in previous publications^22,47^.

### Lung immunohistochemical staining

In the fixed lung tissues, 5-μm sections were cut and deparaffinized. Antigen retrieval was performed in 10 mM citrate buffer, pH 6.0, in a pressure cooker for 10 min. Endogenous peroxidase activity was inhibited using a 3% H_2_O_2_ solution applied to the slides for 15 min, followed by a 30-min blocking step using 3% BSA in PBS. The slides were then incubated with rabbit anti-mouse fibronectin antibody (1:1000, Proteintech) for 1 h at room temperature. The slides were further stained with horseradish peroxidase (HRP)-conjugated goat anti-rabbit IgG (EarthOx Life Sciences, CA, USA) for 50 min at room temperature. Then, freshly prepared DAB chromogenic reagent was added to mark the tissues. Finally, the sections were counterstained with hematoxylin staining solution and captured at ×400 magnification with a microscope (model BX-53, Olympus Optical).

### Immunofluorescence staining

As described in the lung immunohistochemical staining, 5-μm sections were prepared and antigen retrieval was performed. Spontaneous fluorescence quenching reagent was added and incubated for 5 min, followed by a 30-min blocking step using 3% BSA in PBS. The slides were then incubated with rabbit polyclonal antibody against histone 3 (citrulline R2 + R8 + R17) (1:300, ab5103, Abcam) and mouse anti-MPO antibody (1:300, ab90810, Abcam) for 1 h at room temperature. The slides were further stained with Alexa Fluor® 555-conjugated goat anti-rabbit antibody (1:500, Life Technologies, USA) and Alexa Fluor® 647-conjugated goat anti-mouse antibody (1:1000, Life Technologies, USA) for 50 min at room temperature. Then, the slides were incubated with DAPI (YESEN, China) solution for 10 min at room temperature. Images were captured with an Olympus IX73 fluorescence microscope using the appropriate lenses and filters.

For in vitro cell experiments, neutrophils (1 × 10^5^) were seeded on a 35 mm confocal dish with a 14 mm bottom well (D35–14–1-N, Cellvis, China) and incubated for 1 h in a CO_2_ incubator at 37 °C. Then, the cells were stimulated with either 60 ng/ml recombinant IL-33 (rIL-33) (210–33, Peprotech, USA) or an equal volume of PBS for 4 h^17^. After incubation, neutrophils were collected, and fixed in 4% paraformaldehyde. The blocking, antibody incubation, and image acquisition steps for these sections were performed as described for lung tissue slides experiments.

### RNA isolation and quantitative real-time PCR

Total RNA was obtained from fresh lung tissue with a TRIzol reagent kit (Life Technologies) and reverse transcribed into cDNA with a reverse transcription kit (Abmgood, Zhenjiang, China) according to the manufacturer’s instructions. TNF-α, IL-1β, IL-6, IL-33, and β-actin mRNA expression was quantified with a StepOnePlus Real-Time PCR System (ABI, USA). The primers used for real-time PCR were designed by referring to PrimerBank (https://pga.mgh.harvard.edu/primerbank). Primers with the following sequences were used:

IL-33 forward, 5-TCCAACTCCAAGATTTCCCCG-3;

IL-33 reverse, 5-CATGCAGTAGACATGGCAGAA-3;

IL-1β forward, 5-GCAACTGTTCCTGAACTCAACT-3;

IL-1β reverse, 5-ATCTTTTGGGGTCCGTCAAC-3;

IL-6 forward, 5-TAGTCCTTCCTACCCCAATT-3;

IL-6 reverse, 5-TTGGTCCTTAGCCACTCCTTC-3;

TNF-α forward, 5-GACGTGGAACTGGCAGAA-3;

TNF-α reverse, 5-TTGGTGGTTTGTGAGTGTGA-3;

β-actin forward, 5-GGCTGTATTCCCCTCCATCG-3; and

β-actin reverse, 5-CCAGTTGGTAACAATGCCATGT-3.

Relative levels were determined using the 2^−ΔΔCt^ method, and β-actin was used as the internal control^48^. Each sample was run in triplicate, and the results were representative of at least three independent experiments.

### Protein extraction and western blotting

Total protein was extracted from cells or tissues by lysis with RIPA buffer containing protease and phosphatase inhibitor cocktails (Beyotime, Shanghai, China) and sonicated on ice three times for 20 s each time. Protein concentrations were determined with a bicinchoninic acid (BCA) assay. Equal amounts of proteins were used for SDS-PAGE. The proteins were separated by 10% SDS-PAGE and transferred to polyvinylidene fluoride membranes (Millipore, Billerica, USA). The membranes were blocked for 1 h in 5% skim milk at room temperature and incubated at 4 °C overnight with the following primary antibodies: anti-histone 3 (citrulline R2 + R8 + R17, ab5103, Abcam), anti-IL-33 (ab54385, Abcam), anti-Fibronectin (151613–1-AP, Proteintech), anti-β-actin (4970 L, Cell Signaling Technology), and anti-GAPDH (5174 s, Cell Signaling Technology). The membranes were then washed three times with Tris-buffered saline Tween-20 (TBST) and incubated with HRP-conjugated goat anti-rabbit IgG (EarthOx Life Sciences, CA, USA) or goat anti-mouse IgG (H + L) HRP (s0002, Affinity Biosciences) for 1 h at room temperature. GAPDH was used as an internal control. The antibody–antigen complexes were detected with Immobilon Western Chemiluminescent HRP Substrate (Millipore, MA, USA) and visualized using the G:Box gel doc system (Syngene, UK).

### Statistical analysis

Statistical analyses were performed using GraphPad Prism 7.0 software. Values are expressed as the means ± SDs. Differences between the groups were assessed by one-way analysis of variance and the Student–Newman-Keuls test (for multiple comparisons). Statistical significance was defined as follows: *P < 0.05; **P < 0.01; ***P < 0.001; ****P < 0.0001; and NS, not significant.
